# Obesity, maternal smoking and SHBG in neonates

**DOI:** 10.1186/s13098-016-0158-0

**Published:** 2016-07-26

**Authors:** Swapna Dharashivkar, Lawrence Wasser, Richard N. Baumgartner, Jeffrey C. King, Stephen J. Winters

**Affiliations:** 1 Division of Endocrinology, Metabolism and Diabetes, University of Louisville, ACB-A3G11, 550 Jackson Street, Louisville, KY 40202 USA; 2 Department of Pediatrics, University of Louisville, Louisville, KY 40202 USA; 3 Department of Epidemiology and Population Health, University of Louisville, Louisville, KY 40202 USA; 4 Division of Maternal-Fetal Medicine, University of Louisville, Louisville, KY 40202 USA

**Keywords:** Sex hormone binding globulin, Maternal smoking, Obesity, Newborn

## Abstract

**Background:**

Sex hormone binding globulin (SHBG), a glycoprotein produced by hepatocytes that transports testosterone and other steroids in plasma, is a marker for developing metabolic syndrome and T2DM. SHBG is present in umbilical cord blood where it may be epigenetically regulated. This study was conducted to investigate whether the fetal environment, based on maternal pre-pregnancy weight, pregnancy weight gain or smoking during pregnancy, influence SHBG in newborns.

**Methods:**

Maternal and newborn characteristics and SHBG levels and other variables were measured in cord and day 2 heel-stick blood samples in 60 healthy full-term singleton babies (31 F, 29 M).

**Results:**

SHBG levels varied nearly fivefold among male and female newborns and were unrelated to sex, neonatal adiposity, determined by the Ponderal index and skinfold thickness, nor TNF∝ in cord blood. There were also no statistically significant associations between pre-pregnancy weight or pregnancy weight gain and newborn SHBG levels. However, cord blood SHBG was higher and insulin levels were lower when mothers were smokers, but normalized by day 2.

**Discussion:**

While SHBG levels are low in obese children and adults, and portend the development of metabolic syndrome and T2DM, our study of healthy babies born to normal women, found no connection between maternal obesity or newborn adiposity and SHBG levels in newborns. Insofar as women who smoked during pregnancy were thinner and had lower cord blood insulin levels than nonsmokers, higher SHBG in their newborns at birth might have been due to insulin sensitivity, or perhaps to an effect of smoking on placental gene expression.

**Conclusions:**

Factors other than maternal weight and pregnancy weight gain appear to be the major determinants of SHBG in newborns. Higher SHBG levels when mothers smoke during pregnancy may contribute to overweight beginning later in childhood. Whether newborn SHBG levels predict the development of overweight and metabolic syndrome remains to be determined.

## Background

Sex hormone binding-globulin (SHBG) is a circulating glycoprotein secreted predominantly by hepatocytes which binds certain sex steroids, including testosterone, dihydrotestosterone, and estradiol, and regulates their entry into target cells [1]. The SHBG gene, located on chromosome 17 (17p13-1p12), and its protein product are regulated by genetic factors, nutritional cues and various hormones. Notably, SHBG levels in children and adults are inversely related to adiposity and to insulin resistance [2]. SHBG levels are reduced in children born at low birth weight [3], in teenage children with the metabolic syndrome [4], and in pre-pubertal children whose parents have the metabolic syndrome [5]. Moreover, low levels of SHBG in adolescence predict adiposity and insulin resistance in adulthood [6, 7]. Thus there is increasing research on SHBG.

SHBG is present in cord blood where levels vary among individuals by as much as tenfold [8] but appear not to differ by sex [9, 10] or race [11]. Cord blood SHBG levels were inversely related to cord blood insulin in a combined population of healthy pregnant women and those with gestational diabetes, but mean levels did not differ between the two groups [12]. Cord blood SHBG levels were higher when pregnant women with PCOS were treated with metformin than with placebo [13]

The concept of fetal programming has been gaining momentum with increasing evidence that the intrauterine milieu influences disease development later in life [14]. Children born to obese mothers are more likely to become obese [15], and excessive maternal pregnancy weight gain results in higher birth weight infants, which, in turn, is associated with subsequent obesity and obesity-related negative health outcomes [16]. Cigarette smoking during pregnancy is associated with reduced fetal growth and an increased risk for intrauterine growth retardation [17, 18], with increased obesity as early as age 5 years [19], and with increased diabetes risk when babies reach adulthood [20]. In rats, prenatal nicotine exposure produced a marked hypertrophy in adipocytes, and a decrease in pancreatic islet size, along with glucose intolerance and insulin resistance in adulthood [21]. Newborn babies exposed in utero to tobacco smoke have increased cord blood concentrations of prolactin and growth hormone [22] and cortisol [23], but no studies have examined SHBG.

In this study, we sought to determine the impact of the intrauterine environment based on maternal pre-pregnancy weight and pregnancy weight gain, and maternal smoking status, as well as newborn adiposity on the variability in SHBG among neonates born to healthy mothers. Since SHBG is expressed in the placenta, and the mRNA is reduced in mothers with gestational diabetes [24], we studied SHBG in neonates on day 2 as well as in cord blood. This research begins to explore the overall hypothesis that SHBG levels in the fetus and newborn are partly under genetic control but are also regulated by the intrauterine environment, and may contribute to and predict the development of adiposity, metabolic syndrome and diabetes as children grow older.

## Methods

We evaluated mothers without pre-eclampsia, diabetes or complications during pregnancy who gave birth to singleton babies born by vaginal delivery. Informed consent was obtained in accordance with a protocol approved by the Institutional Review Boards of the University of Louisville and Louisville University Hospital. Babies had an Apgar score of 7 or greater at 5 min, and no apparent congenital anomalies. 66 mothers provided informed consent but data were complete for 60 women who gave birth to 31 girls and 29 boys. For 6 babies, blood samples were lost and not analyzed. There were 24 whites, 25 blacks, and 17 babies who were mixed race or other. Maternal pre-pregnancy weight was obtained by chart review or by recall. Gestational weight gain was calculated as the difference between the last weight measured before delivery and the pre-pregnancy weight. Placental weight was measured after removal of the umbilical cord and membranes. Mothers were classified as smokers if they answered ‘yes’ to the question, “have you smoked during your pregnancy?” All participants denied use of alcohol during pregnancy.

Birth weight (gm) and length (cm) were determined, and babies were re-examined on day 2 of age at which time the subscapular skin fold (SSF) and triceps skin fold (TSF) thickness were determined by a pediatrician (LW) using a Harpenden caliper. The Ponderal index, [(weight in gm) × 100/(length in cm)^3^] was calculated as a measure of infant adiposity.

Venous cord blood was collected from a clamped segment of the umbilical cord at delivery, and heel-stick blood samples were obtained during the physical examination on day 2. Glucose was measured using a glucometer (StatStrip blood glucose meter, Nova Biomedical, Waltham, MA, USA). Samples were centrifuged and stored at −20 °C for assay. SHBG and insulin were measured using ELISA kits from Alpco (Salem, NH, USA). TNF∝ was measured using the Quantikine HS ELISA Human TNF alpha assay (R and D Systems, Minneapolis, MN, USA).

Statistical analyses used t tests to compare group means and linear regression to evaluate associations of SHBG levels with continuous covariates. All analyses were conducted using STATA data analysis and statistical software (College Station, TX, USA). Effect modification by smoking was modeled using multiplicative interaction terms in regression analysis. Multiplicative interaction is the most common type of effect modifications; additive interaction, which is rarer, was not modeled.

## Results

SHBG levels in cord blood ranged from 20.5 to 90.8 nmol/L, and in the heel stick sample on day 2 from 15.2 to 80.3 nmol/L. Overall there was no significant difference between the mean (±SD) levels in boys (45.1 ± 18.9) and girls (39.9 ± 10.7 nmol/L) in either cord blood or the samples taken on day 2 (40.4 ± 15.0 vs 37.2 ± 14.1 nmol/L).

**Fig. 1:**
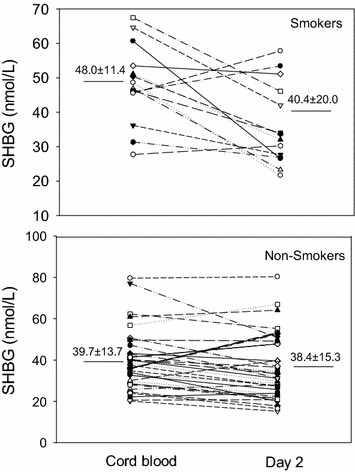
Levels of SHBG in cord blood and heel stick blood in babies born to mothers who smoked during pregnancy and those who did not smoke. *Open symbols* are boys and *filled symbols* are girls. Of the 14 smokers, the result on day 2 was lower in 11. Mean (±) SD values are also shown. The difference between the two samples for babies whose mothers smoked was significantly different when analyzed by paired *t* test (p = 0.0135)

Results for women who smoked during pregnancy (n = 14) and their babies are compared to nonsmokers (n = 46) in Table 1. Mothers who smoked were thinner, and their cord blood had lower insulin and higher SHBG levels than the non-smokers. The average birth weight of babies born to smokers was 93 g lower, which was 4.8 % less (p = NS), than for nonsmokers while placental weights were similar. One baby in the former group and two in the latter group were low birth weight (<2500 g). By day 2, SHBG levels in babies born to smokers declined to the levels of nonsmokers. Figure 1 shows the individual values for SHBG in smokers and non-smokers. In contrast to the babies born to smokers, SHBG levels in the newborns of non-smokers were similar in cord blood and on day 2.

**Table 1:** Clinical characteristics and hormone values in women who smoked during pregnancy and nonsmokers, and their offspring

Variable	Smokers (n = 14)	Nonsmokers (n = 46)	p value
SHBG in cord blood (nmol/L)	48.0 ± 11.4	39.7 ± 13.7	0.04
Cord blood insulin (U/L)	2.0 ± 0.71	3.1 ± 1.94	0.045
Cord blood TNF∝ (pg/mL)	6.92 ± 2.54	7.68 ± 4.22	
Cord blood glucose (mmol/L)^a^	6.32 ± 1.10	5.88 ± 1.81	
SHBG day 2 (nmol/L)	40.4 ± 20.0	38.4 ± 15.3	
Birth weight (g)	3032 ± 402	3184 ± 401	
Placenta weight (g)	487 ± 83	480 ± 111	
Maternal age (years)	25.7 ± 4.7	26.7 ± 6.2	
Maternal pre-pregnancy wt (kg)	60.9 ± 13.0	72.3 ± 19.1	0.03
Pregnancy weight gain (kg)	14.3 ± 7.4	14.3 ± 9.3	

Results are Mean ± SD

^a^Glucose levels were available for 42 subjects

**Fig. 2:**
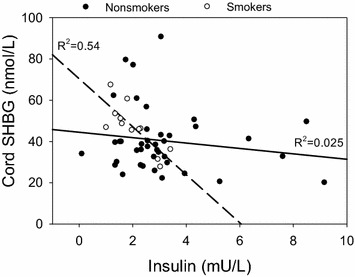
Scatterplot showing the relationship between the levels of insulin and SHBG in cord blood samples among male and female newborns by maternal smoking status. *Interrupted line* indicates the regression plot for smokers (R^2^ = 0.54; p < 0.003), and the *solid line* is for non-smokers. (R^2^ = 0.03; p = 0.26)

Figure 2 shows the relationship between cord blood insulin and SHBG. There was a statistically significant, inverse association of cord blood insulin with SHBG in smokers (R^2^ = 0.54; p < 0.003), but no association in nonsmokers (R^2^ = 0.03; p = 0.26); hence, there was a statistically significant interaction between smoking and the association of cord insulin with SHBG (p = 0.04). Multiple regression analysis suggested that the main effect of smoking on cord blood SHBG and the interaction of smoking with cord blood insulin were independent of pre-pregnancy weight, pregnancy weight gain, race, placental weight, birthweight and skinfold thickness. This finding suggests that maternal smoking modifies the relationship between insulin and SHBG in cord blood regardless of measures of maternal or infant body size and adiposity.

TNF∝ was reported to suppress SHBG production in HepG2 hepatocarcinoma cells by decreasing the level of the transcription factor HNF4∝ [25]. In this study, cord blood TNF∝ levels were not different between smokers and nonsmokers, and were unrelated to cord blood SHBG (R^2^ = 0.03).

**Fig. 3:**
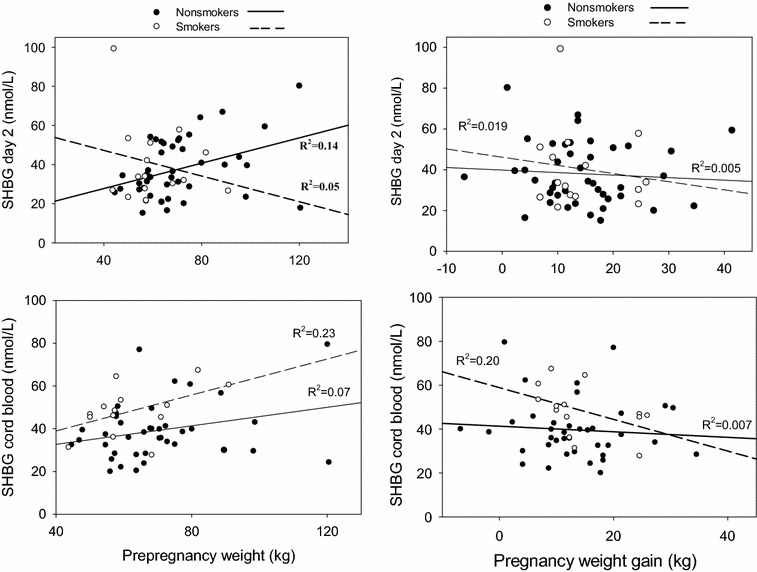
Scatterplots showing the relationship between pre-pregnancy weight and maternal weight gain during pregnancy to SHBG levels in cord blood and in day 2 heel stick samples by maternal smoking status. *Interrupted line* indicates the regression plot for smokers and the *solid line* is for non-smokers

**Fig. 4:**
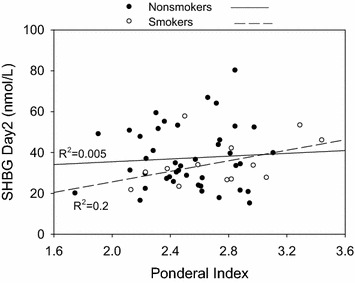
Relationship between the Ponderal index and SHBG in newborn babies on day 2 by maternal smoking status

We next performed linear regression analysis to determine whether anthropometric variables were associated with SHBG (Fig. 3). Neither pre-pregnancy weight nor pregnancy weight gain was an important determinant of SHBG in cord blood. While there was a suggestion for an interaction between smoking and pregnancy weight gain with cord blood SHBG, it was not statistically significant (p = 0.30). Moreover, the apparent inverse association of pregnancy weight gain with cord blood SHBG in smokers (R^2^ = 0.20, p = 0.11) was not altered by adjustment for insulin (R^2^ = 0.198, p = 0.13). Similarly, the association of cord blood SHBG with pre-pregnancy weight (R^2^ = 0.23, p = 0.08) was only slightly diminished by adjustment for insulin (R^2^ = 0.19, p = 0.13). There was no association between pregnancy weight gain and day 2 heel-stick blood samples in either smokers or nonsmokers. In agreement with previous results [26], birth weight was positively related to placental weight (R^2^ = 0.35; p < 0.01; not shown). There was no significant relationship (p > 0.05) between SHBG on day 2 with either subscapular skinfold thickness, triceps skinfold thickness or flank skinfold thickness (not shown), birthweight, or the Ponderal index, a measure of fetal growth and nutrition (Fig. 4).

## Discussion

The gestational period is a crucial time of growth, development and physiological change in the fetus. It is a window of opportunity for intervention via maternal nutrition and/or physical activity that may induce favorable changes in the in utero environment that will influence the lifelong risk for metabolic syndrome and T2DM.

The level of SHBG in plasma is known to be under genetic as well as hormonal and nutritional control. Because SHBG is inversely related to BMI beginning in childhood, and is a marker for insulin resistance and the risk for developing T2DM [1], we hypothesized that SHBG levels at birth might represent a biomarker of fetal metabolic status, and might be related to the newborn’s Ponderal index or skin fold thickness, or might correlate with maternal obesity or pregnancy weight gain. SHBG is expressed in the placenta which might contribute to the level in cord blood; therefore we show, for the first time, SHBG levels in heel-stick blood samples on day 2 as well as in cord blood samples. We recognize, however, that due to a long circulating half-life [27], a small amount of placental SHBG might remain in fetal blood on day 2. SHBG levels in both cord blood and heel stick blood varied among individuals by fivefold, did not differ by sex, and were unrelated significantly to these anthropometric descriptors. In a previous study, SHBG levels were lower in babies born in Boston than in Shanghai where mothers had lower BMI and less pregnancy weight gain [28].

Instead, we discovered higher levels of SHBG in cord blood of maternal tobacco smokers that normalized by age 2 days. It is estimated that 4–25 % of women worldwide continue to smoke during pregnancy, and 24 % of our new mothers viewed themselves as smokers. Maternal cigarette smoking is known to be pathogenic including increasing the risk for prematurity and low birth weight as well as obesity and type 2 diabetes in later life [18]. Mothers who smoked were thinner, and their cord blood insulin concentrations were lower, than for non-smokers. BMI has been higher in maternal smokers than nonsmokers in some studies but not in others [29]. SHBG and insulin levels in cord blood were inversely related among mothers who smoked, but not among non-smokers, suggesting that smoking induces or enhances the relationship between insulin and SHBG. This finding has not been reported previously.

Higher SHBG levels in cord blood of smoking mothers may have resulted from indirect or direct effects of smoking on the fetal liver. To what extent higher SHBG levels in the cord blood of smokers was impacted by their thinness and lower insulin levels is not known. In a previous study, insulin and SHBG levels in cord blood were likewise inversely related among mothers with gestational diabetes [9]. While there is a substantial amount of data supporting an association between higher insulin levels and low levels of SHBG [30], no cellular mechanism has been established for this association, and more recent experiments using hepatocarcinoma cells have challenged the idea that insulin itself is a regulator of SHBG expression [31]. As much as 70 % of the blood supply to the fetal liver is from the umbilical vein, resulting in exposure to high concentrations of xenochemicals from the maternal circulation. In this regard, maternal smoking was found to affect the expression level of a variety of genes in liver samples from fetuses from women undergoing second trimester elective pregnancy termination [32, 33]; however, no information was provided for SHBG.

Higher SHBG levels in cord blood of smokers might instead reflect SHBG of placental origin [24]. Placental DNA methylation patterns are altered with maternal smoking [34], and the expression level of placental genes related to coagulation and vasculogenesis is increased while the expression of cell adhesion-related genes is decreased; however, SHBG was not among the placental genes noted to be differentially expressed [35]. Although no studies have examined the effects of added insulin on placenta SHBG expression, SHBG mRNA levels were lower in placentas from hyperinsulinemic women with gestational diabetes [24]; so it is possible that elevated SHBG in cord blood in smokers in inverse relation to insulin levels was partly from placenta.

Several studies have linked higher maternal pre-pregnancy weight and greater gestational weight gain to adverse perinatal health outcomes including preterm birth, low-birth weight and macrosomia [36]. Moreover, maternal obesity has been associated with offspring with an increased risk for obesity lifelong as well as insulin resistance, hypertension and dyslipidemia, [37]. Higher circulating levels of inflammatory cytokines, insulin resistance and hyperinsulinemia, and dyslipidemia may cause permanent changes in the developing fetus. In one study of women from China, cord blood SHBG levels were lower among babies born to overweight mothers, most of whom had gestational diabetes [38]. In the current study, we found no inverse relationship between maternal obesity or weight gain during pregnancy with SHBG in the newborns of nondiabetic women, and no relationship between SHBG and neonatal markers of obesity including skin fold thickness and the Ponderal index, or to cord blood levels of the cytokine TNF∝. Thus lower SHBG levels born to overweight diabetic women, if confirmed, might be due to diabetes rather than obesity. Moreover, genetic factors may contribute to the substantial variation in SHBG among newborns.

Limitations of this study include a relatively small sample size, especially for women who smoked during pregnancy, the reliance on recall for pre-pregnancy weight, and the broad definition of smoking during pregnancy. Denial of smoking would result in misclassification of smokers as non-smokers. Multiple hormonal and genetic factors are known to influence plasma SHBG levels in adults, but were not studied due to lack of sufficient sample volumes. Finally, intrahepatic fat was reported to be higher in infants born to obese mothers with gestational diabetes compared to infants of nondiabetic normal-weight mothers [39], and hepatic fat is known to be inversely associated with SHBG [40]; however, hepatic fat was not measured in the newborns in this study.

Maternal smoking is one of many factors associated with low birth weight and a subsequent increase in the risk for obesity in childhood and adult life. SHBG levels in low birth-weight boys were not statistically different from controls at age 2–3 months [41], but by age 8 years were lower in low birth weight girls [3], and low SHBG was found with obesity in both sexes by age 6–9 years [42] suggesting that the link between low SHBG and obesity is acquired at a young age. While low SHBG in older children and adults is a recognized marker of insulin resistance and hepatic steatosis, longitudinal studies are needed to determine whether SHBG levels at birth predict metabolic status later in life, and could be used as a marker for early interventions. Studies using human adult liver indicate that the major determinant of hepatic SHBG expression is the level of the transcription factor HNF4∝, a key regulator of hepatocyte function [43]. Whether SHBG is an indicator of HNF4∝ activity in fetal liver, or itself plays a direct role in fetal metabolism and subsequent metabolic health outcomes remains to be determined.

## Abbreviations

SHBG: sex hormone binding-globulin
PCOS: polycystic ovary syndrome
TNF∝: tumor necrosis factor ∝
